# Antenatal group education for pregnant asylum seeker in the netherlands

**DOI:** 10.1016/j.jmh.2026.100401

**Published:** 2026-01-28

**Authors:** Elena Soldati, Anouk E.H. Verschuuren, Joanne Koomans, E.I. Feijen-de Jong, Jelle Stekelenburg, Ineke R. Postma

**Affiliations:** a OLVG (Department of Psychiatry and Medical Psychology), Amsterdam, The Netherlands; b Amsterdam UMC, Vrije Universiteit Amsterdam (Department of Psychiatry), Amsterdam, The Netherlands; c Amsterdam Public Health, Mental Health Program, Amsterdam, The Netherlands; d University Medical Center Groningen & University of Groningen, Department of Health Sciences, Global Health Unit, Groningen, The Netherlands; e Midwife Practice NewLife, Ter Apel, The Netherlands; f University of Groningen, University Medical Center Groningen, Department of Primary medicine and Long-term Care, Groningen, the Netherlands; g Midwifery Academy Amsterdam Groningen, InHolland, Groningen, the Netherlands; h Amsterdam UMC location Vrije Universiteit Amsterdam, Midwifery Science, Amsterdam, the Netherlands; i Frisius MC, Department of Obstetrics & Gynaecology, Leeuwarden, The Netherlands

**Keywords:** Pregnancy, asylum seekers, antenatal group education

## Abstract

**Introduction:**

Pregnant asylum seekers face increased risks of adverse maternal and perinatal outcomes. Group antenatal education may improve access and engagement. We aimed to assess the preliminary impact of implementing antenatal group education sessions for pregnant asylum seekers, focusing on their effects on health literacy and satisfaction with care.

**Methods:**

We conducted a prospective quasi- experimental cohortstudy with pregnant asylum seekers older than 18 years. Participants followed two sessions of group interactive care education next to regular care, while the comparison group received standard obstetric care. Knowledge on perinatal issues, the Dutch healthcare system, and satisfaction of care were tested before and after participating in the sessions. The implementation was assessed following the RE-AIM framework (Reach, Effectiveness, Adoption, Implementation and Maintenance).

**Results:**

Participation in the antenatal group education sessions was associated with an increase in knowledge scores within the intervention group. However, no significant difference was found relative to the comparison group. Satisfaction with care also did not differ significantly between groups. Reach and Adoption showed good results, while Implementation was hampered by the frequent relocations.

**Conclusions:**

A relatively small sample size and high loss to follow-up because of frequent relocations have influenced the results of this study.

## Highlights

• Antenatal group education may improve health literacy, but effectiveness was inconclusive due to small sample size and frequent relocations.
• Frequent relocations limit continuity of care and research, highlighting the need for stronger policies supporting pregnant asylum seekers.

## 1 Introduction

Pregnancy is generally considered a state of potential heightened vulnerability (van der Zande et al. 2017). When someone flees from their country to resettle somewhere safer while pregnant, they are at risk of adverse maternal and perinatal outcomes (Heslehurst et al, 2018; Gieles et al 2019). International research shows that pregnant asylum seekers^1^ face higher rates of perinatal mental health disorders, maternal mortality, preterm birth and severe acute maternal morbidity, compared to the host population (Heslehurst et al 2018; Gieles et al 2019). These health disparities are multifactorial: limited access to maternity health services, social isolation, poverty, communication barriers and experiences of discrimination are some of the social determinants of health that can negatively affect maternal and perinatal outcomes (Heslehurst et al 2018; Rowe et al 2023).

Studies focusing on the Netherlands have found similar results. Pregnant asylum seekers in the Netherlands have increased perinatal and maternal mortality rates compared to the general Dutch population (Verschuuren et al 2020; van Oostrum et al 2011). Multiple health organizations have highlighted that healthcare for asylum seekers in the Netherlands is suboptimal, partly due to restrictive policies (Inspectie Gezondheidszorg en Jeugd 2022; VluchtelingenWerk Nederland 2023; Dokters van der Wereld et al 2023). In 2023, pregnant people had to sleep on a grass field outside one of the central asylum accommodation centers (VluchtelingenWerk Nederland 2023) or were placed in crisis emergency locations where only emergency healthcare was available (VluchtelingenWerk Nederland 2023; Dokters van der Wereld et al 2023). Continuity of care is undermined for pregnant asylum seekers, mainly as a result of frequent relocations (Kasper et al 2022), which cause delays in diagnosis, missed follow-up appointments, loss of preventive care, and more frequent emergency procedures or hospitalizations. These disruptions also reduce trust and engagement with the healthcare system (Cummins et al 2024; Pereira Gray et al 2018). Restrictive policies therefore disrupt healthcare for pregnant asylum seekers and make it difficult for healthcare providers to deliver adequate care. Furthermore, a study showed that discriminatory practices, stemming from either conscious or unconscious bias among healthcare providers, can result in suboptimal care for pregnant immigrants (Jonkers et al 2011; Overtoom et al., 2026). To improve maternity care for asylum seekers in the Netherlands we need programs that facilitate access to and utilization of maternal health services. An alternative healthcare strategy may be group antenatal care. This type of care aims to combine clinical care with health promotion, information, peer support, social network and community building (Sadiku et al 2023). Group antenatal care meetings promote interaction between participants and are led by midwives. The most common form of group antenatal care is CenteringPregnancy (Wagijo et al 2024). In the Netherlands, a study on group antenatal care showed a decrease in adverse maternal outcomes in nulliparous participants (Rotundo 2011). Another study showed how participating in group antenatal care empowers pregnant persons to adopt healthier behaviors such as quitting smoking, exercising and eating healthier, as they gained knowledge on pregnancy and birth (Wagijo et al 2023). These healthy behaviors continued in the postpartum period as well (Wagijo et al 2023). Finally, one study showed how group antenatal care also improved participant perceptions of social support and sense of belonging, as well as a feeling of empowerment and engagement in their antenatal care (Rijnders et al 2019). Previous group antenatal care studies in the Netherlands have excluded pregnant people with insufficient Dutch or English language skills (Rotundo 2011; Wagijo et al 2023; Wagijo et al 2022), although international research suggests that especially migrant populations might benefit from this type of care (Feijen-de Jong et al 2015). A systematic review (2024) on maternity care for pregnant refugees and migrants in high-income countries included nine articles on group antenatal care (Yeshtila et al 2024). Four of those studies were performed in the USA (Liu et al., 2017; Torres et al 2012; Madeira et al 2019; Reavy et al 2012), three in Australia (Owens et al., 2016; Riggs et al 2021; Dube et al 2023), one in Canada (Hetherington et al 2018) and one in the UK (Rayment-Jones et al 2021). Group antenatal care reduced adverse psychological symptoms (Riggs et al 2021) and birth complications (Dube et al 2023). It increased the breastfeeding rate (Torres et al 2012), enhanced continuity of care (Dube et al 2023), health literacy, health advocacy, self-efficacy (Torres et al 2012; Madeira et al 2019) and a sense of belonging and support (Owens et al., 2016; Riggs et al 2021). The review concluded that group antenatal care improved health outcomes and satisfaction with care (Yeshtila et al 2024). Another systematic review (2025) aimed at assessing perinatal mental health interventions for pregnant refugees, showed that group sessions can improve patient satisfaction and reduce stress, depression, and social isolation (Ether et al 2024). Both reviews agreed that further research is needed to explore alternative antenatal interventions for refugee populations, using appropriate and replicable measures, to adapt policies and improve maternity care for this group. There are some initiatives in the Netherlands providing group antenatal care programs (CenteringZorg 2025). However, to date, no study has examined the effect of group antenatal care on health literacy and health satisfaction among pregnant asylum seekers in the Netherlands.

This study aims to evaluate the preliminary effects of antenatal group education sessions for pregnant asylum seekers on maternity health literacy and perceived satisfaction with care and secondly to evaluate the implementation of the group education program during this pilot study. Participants will complete questionnaires about maternal health and satisfaction of care. We hypothesize an improvement in scores between before and after participating in the sessions and between participants in the intervention group and in the comparison group. Box 1

**Box 1:** definitions of terminology used in the text.

Asylum-seeker: ‘An individual who is seeking international protection. In countries with individualized procedures, an asylum-seeker is someone whose claim has not yet been finally decided on by the country in which the claim is submitted. Not every asylum-seeker will ultimately be recognized as a refugee, but every refugee was initially an asylum-seeker.’ (UN General Assembly 1951) Refugee: ‘a person who owing to a well-founded fear of being persecuted for reasons of race, religion, nationality, membership of a particular social group, or political opinion, is outside the country of this nationality and is unable to or, owing to such fear, is unwilling to avail himself of the protection of that country’ (UNHCR, 2006). Migrant: While there is no formal legal definition of an international migrant, most experts agree that an international migrant is someone who changes his or her country of usual residence, irrespective of the reason for migration or legal status (United Nations, n.d).

## 2 Methods

### 2.1 Design

A prospective quasi- experimental cohort study was conducted among pregnant asylum seekers at the asylum seekers’ center in Ter Apel. This center is one of the two central locations in the Netherlands where every asylum seeker entering the country is accommodated initially. The STROBE (Strengthening the reporting of observational studies in epidemiology) guidelines were applied for the reporting of this study (von Elm et al 2007). The RE-AIM (Reach, Effectiveness, Adoption, Implementation, Maintenance) framework was used to guide the evaluation of the implementation process and outcomes (Glasgow et al 2019).

From February 2017 to December 2018, antenatal group education sessions were offered at this location, as part of a ZonMW subsidized pilot project (Healthcare Research Nederland, Medical Science; Dutch abbreviations). While Centering Pregnancy offers antenatal group education sessions combined with regular care; it was decided prior to the start of the project by the team to separate the sessions from the regular care for this study. As the continuity of care is already hindered in this population, due to relocations, we decided not to interfere with their regular care. Therefore, only the group education activity of the Centering Pregnancy model was implemented in the study, while the individual health assessments were performed as standard care. Afterwards, a comparison group of women, who received regular antenatal care, was included until December 2023. The recruitment of the comparison group happened after the pilot project was finished, instead of simultaneously, since an asylum seeker center is an enclosed environment, and therefore participants receiving regular care might have been influenced by participants receiving antenatal group education.

Different local organizations were involved in the development of the antenatal group education project: the midwifery practice in Ter Apel (NewLife), the hospital in Stadskanaal (Treant ZorgGroep), the municipal health service (GGD Groningen), a maternity care assistants organisation (Kraamzorg Groene Kruis) and an advisory organization for primary care professionals ELANN. In addition, the Healthcare Center for Asylum Seekers of Ter Apel (GZA; gezondheidszorg asielzoekers), the Rutger Knowledge Center and Pharos expertise center on healthcare disparities, asylum seekers and low-literacy were consulted during the project. Researchers from the University Medical Center Groningen (UMCG) were involved in assessing the results of this project by means of this pilot study.

### 2.2 Ethics

This study was reviewed by the accredited medical ethical committee of the University Medical Center Groningen (UMCG). The committee declared that the study was not subject to the Dutch Medical Research Involving Human Subjects Act (WMO).

### 2.3 Participants

All pregnant asylum seekers aged 18 years or older in Ter Apel were eligible to participate in the antenatal group education program. They provided written informed consent in English in the presence of an official interpreter to participate in the study. Participants were recruited using consecutive convenience sampling during routine prenatal visits. Only those who agreed to participate and provided written informed consent were included; no random sampling was applied. Therefore, the sample represents those who consented rather than all pregnant asylum seekers. A female coordinator contacted the participants, planned, and hosted the meetings.

### 2.4 Intervention

Participants for the intervention were recruited using a convenience consecutive sampling from all eligible pregnant asylum seekers. The initial design of the antenatal group education program consisted of three sessions, each separated by one week. However, due to the frequent relocation of asylum seekers, early in the inclusion phase, this model proved infeasible. Therefore, the program was changed to two group education sessions, which combined the content from the second and third sessions.

The groups consisted of a minimum of five and a maximum of ten participants. Participants attended these sessions without their partners, friends, or relatives. Where possible, groups consisted of participants who spoke the same language, and the same female interpreter was present at all sessions. These sessions were held in addition to the regular midwife visits and were led by a registered midwife (from NewLife midwifery practice). All sessions were led by the same facilitator. The sessions were organized every week. We aimed to create a group feeling among the participants, so that the interactive part would continue outside the sessions. The content of the sessions was tailored to accommodate different levels of knowledge, ranging from basic to more advanced topics, in response to the needs of the participants. Furthermore, the Dutch medical and obstetric system was explained in detail, as the participants were unfamiliar with it. The use of an in-person interpreter helped bridge language and cultural barriers. Sensitive topics, such as abortion, contraception, pregnancy, and birth, were discussed in a culturally sensitive way by inviting participants to share their opinions and customs without making assumptions and explaining reproductive healthcare rights in the Netherlands. The content of the two sessions is presented in Appendix 1. The content of these sessions was based on topics considered necessary from the midwives working with pregnant asylum seekers, as well as conversations previously had with pregnant asylum seekers.

### 2.5 Comparison group program

Participants in the comparison group were recruited at the midwifery clinic in Ter Apel during routine prenatal visits, after the intervention period had ended. Sequential recruitment was chosen to avoid contamination within the enclosed asylum center environment, where participants receiving regular care might otherwise have been influenced by those attending antenatal group education sessions. We aimed to include participants who matched those of the intervention group based on specific criteria: same country of origin, gestational age and parity. We initially aimed to perform 1:1 matching of comparison participants to intervention participants based on country of origin, gestational age, and parity. Eligible participants were approached by the midwife and invited to participate in the study. Inclusion criteria were identical to those of the intervention group, except for enrollment in the antenatal group education program. However, due to limited comparison group recruitment (due to high turnover/relocations at the center) it was not feasible to achieve successful individual matching. Therefore, matching was not successful.

### 2.6 Data collection

The level of knowledge regarding pregnancy, birth, and the Dutch healthcare system of the participants was assessed using a knowledge questionnaire (Appendix 3). This questionnaire was based on the TNO questionnaire of the Connect-In study evaluating the effects of Centering Pregnancy (Wagijo 2022). We added 11 questions to the questionnaire based on the KNOV (Royal Dutch Organization of Midwifery) emergency card (Koninklijke Nederlandse Organisatie van Verloskundigen, n.d.) and the Dutch maternity care system.

The intervention group completed the questionnaires at T1 during a regular visit with the midwife before the interactive group education sessions and at T2, after the second antenatal group education meeting. Before the start of the first session (T1), the midwives checked whether the knowledge questionnaire had been filled in. If this was not the case, participants were given the opportunity to complete it before the session began. The comparison group filled in the knowledge questionnaire during regular visits with the midwife. The knowledge questionnaires were checked for correct answers and scored accordingly by the second author. A correct answer scored one; an incorrect or ‘I don’t know’ response scored zero. Total scores went from zero to a maximum of 21.

**Figure 1:**
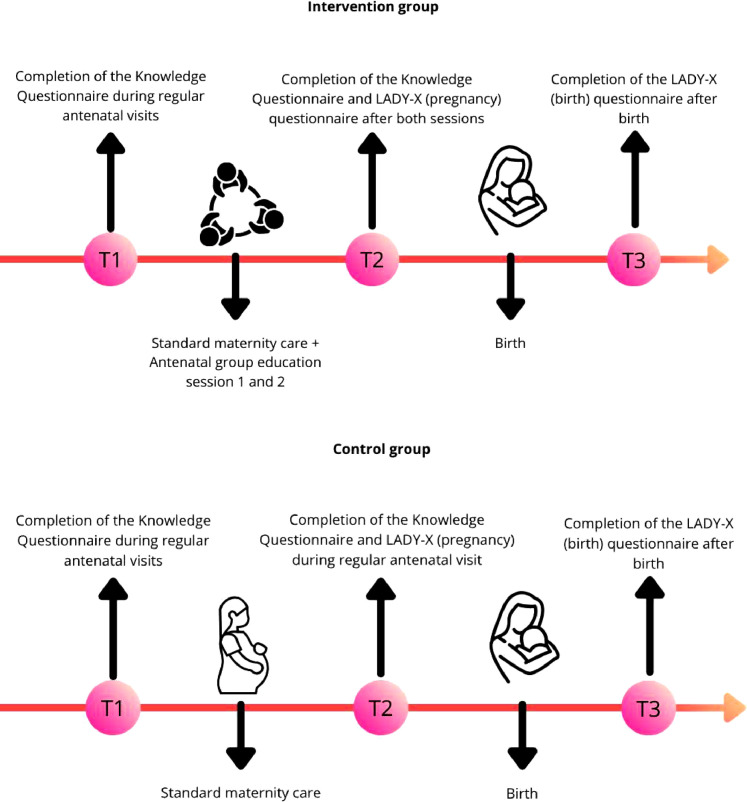
timeline of intervention and data collection.

Patient satisfaction was measured using the validated LADY-X questionnaires (Gartner et al 2015); one concerning pregnancy and one concerning birth (Appendix 4)*.* Each question on the LADY-X can be answered with zero (not satisfied), one (satisfied) and two (very satisfied). The pregnancy LADY-X, total score ranges from zero to ten; while the one on birth went from zero to 14. The LADY-X results were collected for both groups at T2 and at T3 (after birth). See also figure 1 for a visual representation of the intervention and moments of data collection.

All questionnaires were translated by certified translators and were checked for appropriateness by a medical doctor affiliated with organization Pharos, who had that specific language as their first language. This translation process followed the guidelines of Cross Cultural Adaptation of Self-Report Measures from Beaton et al (Beaton et al 2000). The questionnaires took approximately 20 minutes to complete. The questionnaires were administered in the patient’s own language, with the help of an interpreter if necessary. Baseline characteristics were recorded, including education, gravidity, parity, country of origin, and gestational age at the first session.

Implementation was evaluated using the RE-AIM framework (Reach, Effectiveness, Adoption, Implementation, Maintenance). Reach was assessed as the proportion of eligible women who participated compared to the number of live births recorded in the same period in Ter Apel. Effectiveness was evaluated via the questionnaire outcomes intra groups and inter group. Adoption was measured as the number of professionals and organizations involved in delivering the intervention. Implementation was assessed through consistency in follow-up. Maintenance was not evaluated, as the preliminary phase of this pilot project was not suited for this purpose.

### 2.7 Statistical analysis

Since this study is a pilot project, we did not perform a sample size calculation.

#### 2.7.1 Baseline Characteristics

Descriptive statistics were used to identify differences in baseline frequencies and characteristics between the groups. Gestational age, years of education, gravidity and parity were all continuous numerical variables; first, the normality was assessed via the Shapiro Wilk test. Depending on their normality the variables were described using either mean and standard deviation or median and interquartile range. Finally, data were compared using either unpaired T-test or the Mann Whitney U test. Descriptive statistics were used to showcase the country of origin of the participants in both groups.

#### 2.7.2 Knowledge questionnaires

The number of correct answers was summed and assigned a total as a discrete numerical variable ranging from zero to 21. Afterwards, its normality was assessed with the Shapiro Wilk test.

Comparison between the total score at T1 and at T2 within the intervention and the comparison group was assessed with either the paired t-test or the Wilcoxon test, depending on normality. Inter-group comparisons at T1 and at T2 between the intervention and comparisongroups were analysed via the unpaired t-test or via the Mann-Whitney U test, depending on normality.

#### 2.7.3 Lady X questionnaires

The total score of the questionnaires was calculated and assigned a total score as a discrete numerical variable ranging from zero to 10 at T2 and zero to 14 at T3. Afterwards the normality was assessed via the Shapiro Wilk test. Inter-group comparisons at T2 and at T3 between intervention and comparison group were analysed via the unpaired t-test or via the Mann-Whitney U test depending on normality.

#### 2.7.4 Regression analysis

We performed a multiple linear regression analysis with the total knowledge questionnaire score at T2 being the dependent variable and the total score at T1 and group allocation (intervention or comparison group) as independent variables. We repeated this analysis, adjusting for covariates, by adding gestational age, years of education and parity as additional independent variables in a second model.

To investigate whether there is an association between following the sessions and a higher score on the LADY-X at T2 and at T3, two multiple linear regression analyses were performed. In these regressions, the total scores at T2 and T3 served as the dependent variables. In both cases, the first model included group allocation (intervention or comparison group) and baseline score as independent variables, while the second model was additionally adjusted for demographic characteristics (gestational age, years of education and parity).

Although the knowledge score and LADY-X score represent a discrete count, we used linear regression as an exploratory approach without assuming normality of the outcome. Residual diagnostics were inspected to check for major violations. This choice was pragmatic given the pilot nature of the study.

#### 2.7.5 Implementation

RE-AIM indicators were analyzed descriptively. Reach, Adoption, and Implementation outcomes were summarized as proportions and frequencies.

## 3 Results

A total of 130 participants were recruited over six years (2017 to 2023); of which 93 took part in the intervention and 37 in the comparison group. The participants in the intervention group were included from February 2017 to December 2018. The antenatal group education sessions were provided in Tigrinya, Arabic and English. A total of 20 groups took place with the amount of participants varying from three to 15 participants. The inclusion of the comparison group continued until December 2023. It was decided to stop the inclusion of the comparison group at that moment since it proved to be difficult to include sufficient participants to match the intervention group who could fill out the questionnaires at both time points, mainly due to the frequent relocations. Frequent relocations refer to the policy-driven transfers of asylum seekers between reception centers across the Netherlands, often occurring multiple times during pregnancy (Tankink et al 2021).

**Figure 2:**
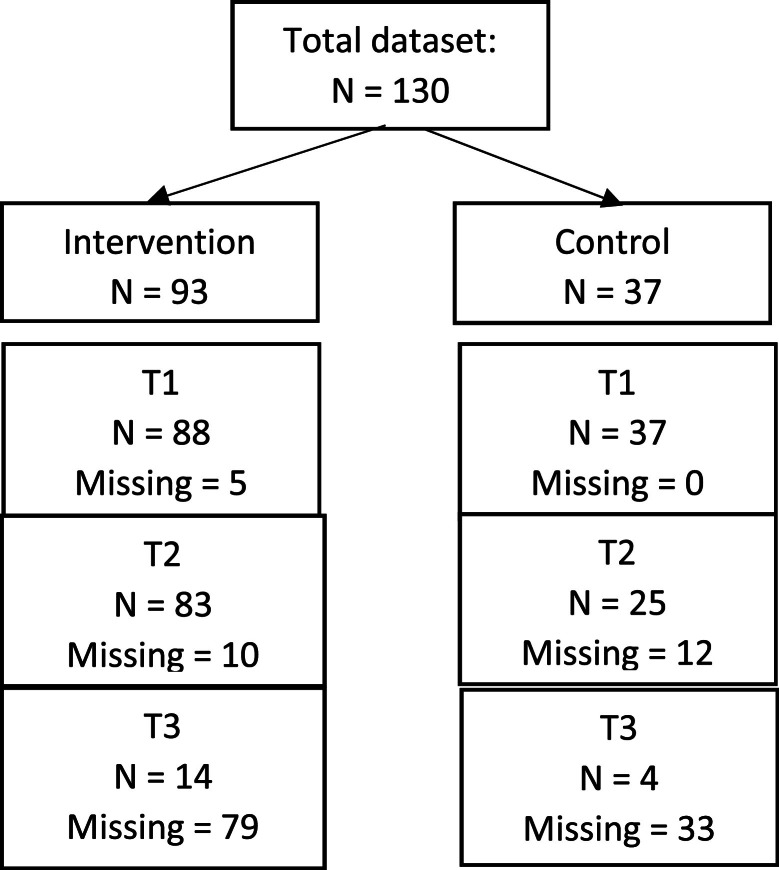
flowchart of missing data at different time points.

Of the total 130 participants missing data were observed at each time point. At T1 (based on knowledge questionnaire), five participants from the intervention group and zero from the comparison group did not complete the questionnaires. At T2 (based on knowledge questionnaire), ten participants in the intervention and 12 in the comparison group were missing. At T3 (based on the LADY-X), 79 participants in the intervention group and 33 in the comparison group were missing. All available data were used in the analyses, with missing values handled as such, in order to preserve the sample size. Figure 2 illustrates the flow of participants and missing data over time.

### 3.1 Baseline characteristics

**Figure 3:**
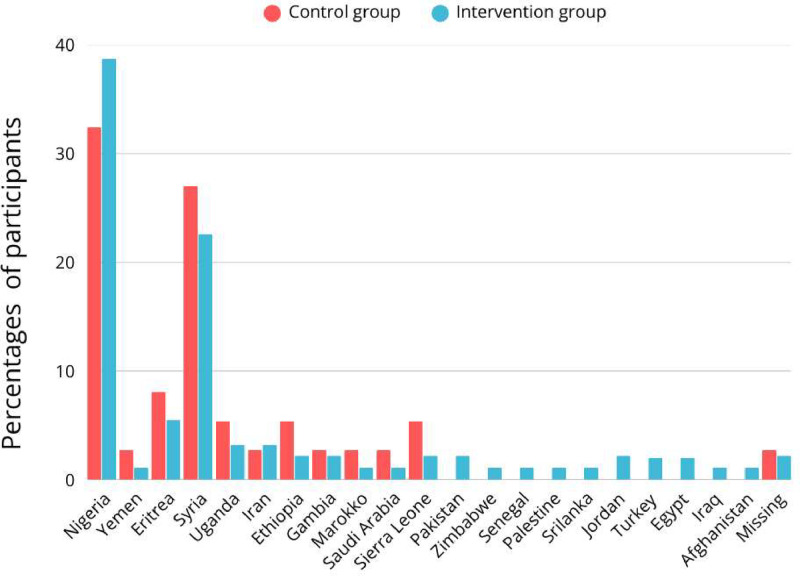
country of origin distribution, comparison vs intervention group.

The years of education differed significantly (P = 0.020) with a median of 12 (IQR 23) in the intervention group and 8 (IQR 21) in the comparison group (Table 1). There was no significant difference between median gestational age (P = 0.091), gravidity (P = 0.295) and parity (P = 0.254) and country of origin (Table 1, Figure 3). There were some missing data regarding the years of education within the intervention group (38) and regarding parity within the comparison group (17).

**Table 1:** Demographic characteristics.

	Intervention N= 93 (71.5%)	Comparison N= 37 (28.5)	p-value
Gestational age^a^*median (range)*	Missing (9) 29 (35)	31 (21)	0.091^a^
Years of education *median (range)*	Missing (38) 12 (23)	Missing (6) 8 (21)	0.020^a^
Parity *median (range)*	Missing (2) 1 (5)	Missing (17) 0 (3)	0.254^a^
Gravidity *median (range)*	Missing (2) 2 (8)	Missing (1) 2 (5)	0.295^a^

a Mann Whitney U test

### 3.2 Knowledge questionnaire inter-group analysis

The results of the total score of the knowledge questionnaire showed a non-normal distribution. Statistical tests were performed with Mann Whitney U test. Both at T1 and at T2 there was no significant difference in the scores found between the groups. In the comparison group, data from 12 participants was missing at T2. The confidence intervals for the estimated differences between medians at both T1 and T2 included zero, indicating no statistically significant differences between the intervention and comparison groups. Table 2, Table 3, Table 4, Table 5

**Table 2:** Knowledge questionnaire inter-group.

	Intervention N= 93	Comparison N= 37	Estimate difference between medians	p-value
T1 Median (range)	Missing (5) 10 (13)	Missing (0) 11 (11)	Estimate = 1.000 CI = 0.000 – 2.000	0.157^a^
T2 Median (range)	Missing (10) 13 (14)	Missing (12) 12 (13)	Estimate = 0.000 CI = -2.000 – 1.000	0.610^a^

a Mann Whitney U test

**Table 3:** Knowledge questionnaire within groups.

	T1	T2	Estimate difference between medians	Negative ranks W^−^	Positive ranks W^+^	p-value
Comparison Median (range)	N= 37 11 (11)	N= 25 12 (13)	Estimate =1.000 CI = 0.000 – 2.500	5	13	0.054
Intervention Median (range)	N= 88 10 (13)	N= 83 13 (14)	Estimate =2.500 CI = 1.500 – 3.000	9	60	<0.001

**Table 4:** Multiple linear regression knowledge questionnaire.

T1 total score	**First model**^a^			**Second model**^b^		
	**B**	**95% CI**	**p-value**	**B**	**95% CI**	**p-value**
	0.637	0.452 – 0.822	< 0.001	0.527	0.283 – 0.771	<0.001
Intervention/ comparison group	0.927	-0.322 – 2.175	0.144	1.556	-0.156 – 3.268	0.074

a Adjusted for: results in T1 and group participation

b Adjusted for: results in T1, group participation, years of education, para, gestational age.

**Table 5:** LADY-X inter-group comparison at T2.

	Intervention N= 93	Comparison N= 37	Estimate difference between medians	p-value
T2 Median (range)	Missing (51) 9 (7)	Missing (16) 8 (4)	Estimate = -1.000 CI = - 1.000 – 0.000	0.113^a^

a Mann Whitney U test

### 3.3 Knowledge questionnaire within group at T1 and at T2 analysis

The results of the knowledge questionnaire were also tested within group comparing the result at T1 with T2 with the Wilcoxon test. Within the comparison group there was no significant difference between T1 and T2 (P = 0.054, CI = 0.000 – 2.500) with a small positive rank value (13). Within the intervention there was a significant difference between T1 and T2 (P < 0.001, CI = 1.500 – 3.000) with a positive rank value of 60 (a stronger and consistent improvement between T1 and T2). Therefore, the score of the knowledge questionnaire at T2 showed improvement compared with the score at T1 in the intervention group.

### 3.4 Multiple linear regression knowledge questionnaire analysis

We used multiple linear regression to examine whether being in the intervention group was associated with higher knowledge scores at T2, while controlling for participants’ baseline scores at T1. Being part of the intervention group (P = 0.144, CI = -0.322 – 2.175) did not significantly contribute to a higher score at T2. The score of the intervention group was 0.927 points higher than the comparison group. A second multiple linear regression analysis was performed correcting for demographic factors in addition to the score result at T1 and being part of the intervention group. This second model also showed no difference between the intervention group and the comparison group, with no statistical significance (P = 0.074, CI = -0.156 – 3.268). The score of the intervention group was 1.556 points higher than the comparison group. Therefore, there is no significant association between an improvement in the score of the knowledge questionnaire and having followed the antenatal group education session.

### 3.5 LADY-X inter-group comparison at T2 analysis

The data of the Lady-X (Cronbach alpha 0.624) questionnaire did not present a normal distribution and the Mann Whitney U test was used to determine a difference between the intervention and the comparison group at T2. There was no significant difference between the two groups at T2 (P = 0.113 CI = -1.000 – 0.000). However, there were many missing data as in the intervention group, 51 participants (54.84%) did not fill in the questionnaire and in the comparison group 16 participants (43.24%) did not complete it.

### 3.6 LADY-X between intervention and comparison at T3 analysis

Due to the frequent relocation of participants after birth, there were not enough participants to perform a statistical analysis, as 33 (89.19%) participants were missing in the comparison group and 79 (84.95%) were missing in the intervention group.

### 3.7 Implementation results

Reach was reflected by the number of included participants in the intervention group (93) compared to the total number of live birth in that same period (125). The reach was therefore 74.4%. Adoption was demonstrated by the involvement of multiple healthcare and support organizations, including the local midwifery practice, hospital, municipal health service, maternity care organization, advisory bodies, and academic partners, all of whom contributed to the delivery and evaluation of the intervention. Implementation was hampered by the frequent relocations, which caused a relatively high attrition rate. In the intervention group between baseline and T1 the attrition rate was 5.4%, between baseline and T2 was 10.8% and between baseline and T3 (postpartum) was 84.9%. The largest loss occurred between T2 and T3. There was a significant difference in years of education between participants who dropped out (M = 10.78, SD = 4.65) and those who completed the study (M = 14.60, SD = 6.93), p=0.044.

## 4 Discussion

This study aimed to assess whether antenatal group education would improve health literacy and perceived healthcare satisfaction in pregnant asylum seekers. Given the small sample size, and high loss to follow-up, findings regarding effectiveness should be interpreted as exploratory. The improvement was measured by comparing the total scores of questionnaires on maternal health literacy and care satisfaction before and after taking part in the group sessions and between the intervention group and the comparison group who followed standard care.

Our study shows a significant improvement in the total score of the knowledge questionnaire after taking part in the antenatal group education sessions in the intervention group. However, there was no significant difference in total score when comparing it to the comparison group. A multiple linear regression analysis revealed no significant association between the intervention and the total score at T2, when corrected for the total score at T1. Regarding satisfaction of care no significant difference was found. However, more than half of the data was missing for the LADY-X in both the intervention and the comparison group. From a RE-AIM perspective, this study demonstrated good Reach, with nearly three-quarters of pregnant asylum seekers participating, and strong Adoption through multidisciplinary collaboration across healthcare and support organizations. Effectiveness outcomes should be interpreted cautiously as elucidated above. Implementation was substantially challenged by frequent relocations, resulting in high attrition and limiting sustained exposure to the intervention as well as outcome assessment.

### 4.1 Interpretations

With regard to improved health literacy, our results are partially in line with previous literature on antenatal group education in pregnant asylum seekers, or people with a refugee or migrant background. Madeira et al (2019) showed that pregnant Somali participants of their group maternity care program improved self-reported health literacy regarding health behaviors. Reavy et al (2012) and Torres et al (2012) showed that antenatal group education improves the knowledge of the healthcare services offered in the host country. Liu et al (2017) showed a higher reported satisfaction of care in marginalized pregnant participants after taking part in antenatal group education. In the general pregnant population, antenatal group education leads to more satisfaction of care, as demonstrated by the systematic review from Sadiku et al (2023). The discrepancies between these studies and our study likely stem from our small dataset and the high number of data missing. In fact, we were not able to analyze the self-reported satisfaction in care after delivery (T3) since 86.15% of participants were not available because they were relocated. Therefore, a direct comparison for satisfaction of care with other studies it is not possible.

The high attrition rate was caused by the constant forced relocations our participants had to undergo due to the Dutch asylum seeker’s polices. Beyond being a methodological limitation, this also highlights an important contextual barrier: frequent relocations undermine the stability required for participants to benefit from sustained group education and for researchers to capture its long-term effects.

Finally, a study in which marginalized pregnant people, who joined an antenatal group education program, were both interviewed and filled in surveys after birth, showed that participants felt more empowered and could better advocate for their needs thanks to the meetings (Liu et al 2017). However, the authors showed that they often lacked continuity of care, which actually undermined their satisfaction of care during and after birth (Liu et al 2017). Our study underscores that, while antenatal group education has the potential to foster empowerment and improve health literacy, its effectiveness depends heavily on the structural conditions in which it is implemented. A recent Dutch study showed that when scientists try to implement health promotion interventions, but ignore institutional or governmental root causes that hold these health inequalities in place, then scientists are not tackling health inequalities, but actually perpetuating them (Dijkstra et al 2025). Therefore, without addressing structural barriers and restrictive policies, antenatal group education programs cannot fully improve health literacy or empower marginalized pregnant participants.

### 4.2 Strengths and limitations

Our study has several strengths: this study is set in the main reception center for asylum seekers entering the Netherlands, which is a unique setting. Asylum seekers centers are secluded locations where group interaction between participants can develop quickly during a program such as antenatal group education. Therefore, implementing such programs in asylum seeker centers can quickly improve the health literacy and health satisfaction of participants, as well as a sense of belonging and community as a previous study has showed (Rijnders et al 2019).

The design of our study presents various strengths, which enhance its acceptability and relevance. As patients with lived experience were included while drafting the content of the sessions, the sessions were patient-centered and patient-tailored. During the group education sessions, we made use of interpreters, had the same leader for every session, provided multidisciplinary knowledge and alternated theoretical parts with more interactive and practical ones. This design is in line with literature concerning recommendations for clinical interventions for pregnant asylum seekers in high income countries (Stevenson et al 2024; Hong et al 2021). Finally, the satisfaction of care was tested with a standardized questionnaire; having a standardized questionnaire enhances this study’s replicability (Gartner et al 2015).

Although we applied the RE-AIM framework to enhance transparency in reporting implementation outcomes, other indicators such as acceptability, fidelity, sustainability signals, and equity implications could not be systematically assessed due to the retrospective nature of this evaluation and feasibility constraints in the asylum seeker setting. Future studies should incorporate these measures prospectively to provide a more comprehensive implementation analysis.

Another limitation concerns the non-concurrent data collection. Since the comparison group was recruited in a different period than the intervention group, the results regarding satisfaction of care may be different due to other cofounders, such as restricted access to care during the Covid-19 pandemic.

The main limitations of our study are the small sample sizes and high loss to follow-up; our results are therefore to be interpreted with caution. The discrepancy between the size of the intervention group and comparison group is also an important limitation. Despite the large period to recruit the comparison group (6 years), only 37 asylum seekers were recruited. This is partially due to a low influx of asylum seekers in the Netherlands during the Covid-19 pandemic (Centraal Bureau voor de Statistiek, 2019), frequent relocations as well as a switch of priority within healthcare during the pandemic.

Other limitations include missing data regarding the demographic information of the participants such as years of education. Due to relocations, it was impossible to contact the participants for whom information was missing in their demographic questionnaires. The loss of participants is representative of the current Dutch asylum seeker’s policies, which puts pregnant asylum seekers at risk for frequent relocations. A previous study which followed pregnant asylum seekers in the Netherlands for five years (2016-2020) showed that almost 70% of them had been relocated at least once during pregnancy (Tankink et al 2021).

### 4.3 Conclusions

This pilot study suggests that antenatal group education may improve health literacy among pregnant asylum seekers; however, these findings are exploratory and should be interpreted with caution due to small sample size and high attrition. Using an implementation lens, the study demonstrates that group education sessions are feasible when supported by interpreters and multidisciplinary collaboration, but require adaptation to address contextual challenges such as frequent relocations. This study highlighted how frequent relocations hinder both access to adequate healthcare and the successful implementation of research. For group education to be effectively introduced and evaluated, stronger asylum and healthcare policies that uphold the health rights of pregnant asylum seekers are essential. Ensuring continuity of care and minimizing relocations are crucial steps toward providing adequate care and enabling meaningful research.

Considering the current political Dutch and Global climate, it is of utmost importance that healthcare providers and scientist strive to create safer environment within the healthcare system and offer quality, continuity and access to care for pregnant asylum seekers (Savas et al 2024). Future research should prioritize implementation-focused designs, such as pragmatic trials or stepped-wedge approaches, to evaluate both effectiveness and feasibility in similar high-mobility settings.

## Fundings

The project received funding from ZonMw (ZonMw; ZorgOnderzoek Nederland + Medical Sciences (Dutch abbreviation: MW).

## Contributors

COA, the midwifery clinic in Ter Apel (NewLife), the hospital in Stadskanaal (Treant zorggroep), the municipal health service (GGD Groningen), the maternity care (Kraamzorg Groene Kruis) and advisory organisation ELANN (ELANN; Nascholing Ondersteuning Huisartsen) worked together to start this program.

## Data availability

The supporting data of this study is available upon request and with a data sharing agreement.

## CRediT authorship contribution statement

**Elena Soldati:** Writing – original draft, Methodology, Formal analysis, Data curation, Conceptualization. **Anouk E.H. Verschuuren:** Writing – review & editing, Methodology, Investigation, Funding acquisition, Data curation, Conceptualization. **Joanne Koomans:** Writing – review & editing, Resources, Project administration, Investigation, Funding acquisition, Data curation. **E.I. Feijen-de Jong:** Writing – review & editing, Supervision, Project administration, Methodology, Data curation, Conceptualization. **Jelle Stekelenburg:** Writing – review & editing, Supervision, Methodology, Investigation, Funding acquisition. **Ineke R. Postma:** Writing – review & editing, Supervision, Project administration, Methodology, Investigation, Funding acquisition, Data curation, Conceptualization.

## Declaration of competing interest

The authors declare that they have no competing interests.

## Figures and Tables

**Table:** 

**1st Meeting (90 minutes)**		
Midwife from midwifery practice	Introduction (20 min)	- Explaining the antenatal group education program - Introduction of participants and their pregnancies - Introduction of leaders of the meeting - Dutch obstetric healthcare system
Midwife from midwifery practice	Informative (15 min)	- Magnetic board with pictures - Regular antenatal care scheme - Caregivers with different genders - Accessibility and contact details caregivers - Medical examinations - Food, vitamins, and lifestyle (smoking, alcohol, drugs)
Midwife from midwifery practice	Interactive (15 min)	- Culture and personal wishes - Antenatal care in the land of birth - Opinion about the Dutch system
COA employee	Informative (15 min)	- Transport - Childcare during delivery - Overnight stay partner - Transfer and procedure after 34 weeks - COA Moneycard
Midwife from midwifery practice	Interactive (15 min)	- Shopping list for the baby - Alarm signal list KNOV
Midwife from midwifery practice	Closing (10 min)	- Questions and discussion

**Table:** 

**2^nd^ + 3^rd^ Meeting (180 minutes)**		
Midwife from midwifery practice	Interactive (20 min)	- Feedback 1st meeting and basket with questions - Examples of when to call whom during deliver
Midwife from midwifery practice	Informative (15 min)	- Movie vaginal birth (animation) - Movie pain relief (animation)
Midwife from midwifery practice	Interactive (15 min)	- Demonstration manikin, pictures, questions and discussion of own experiences
Midwife from hospital	Informative (15 min)	- Induction of labour (pictures, movie and manikin) - Caesarean section (movie)
Midwife from hospital	Interactive (15 min)	- Own culture and wishes - Vaginal birth, caesarean section and induction of labour in country of birth and opinion about the Dutch system
Midwife from midwifery practice	Interactive (10 min)	- Feedback 2nd meeting and basket with questions - Examples of when to call whom during delivery
COA employee	Informative (5 min)	- Transport - Registration of birth at town hall - Children health clinic (vaccination) - Transfer
Midwife from midwifery practice	Informative (5 min)	- Role midwife postpartum - Medication - Postpartum care
Maternity care nurse	Informative (5 min)	- Role of maternity care (information, movie) - Breastfeeding and bottle feeding
	Interactive (15 min)	- Health problems child and problems breastfeeding - Demonstration breastfeeding
Municipality health educator	Informative (15 min)	- STD education (educational material) - Sexuality (educational material) - Contraception (contraception case)
Municipality health educator	Interactive (15 min)	- Opinions and knowledge about contraception and STD’s, gender in sexuality
Midwife from hospital	Closing (10 min)	- Questions and discussion

**Figure:**
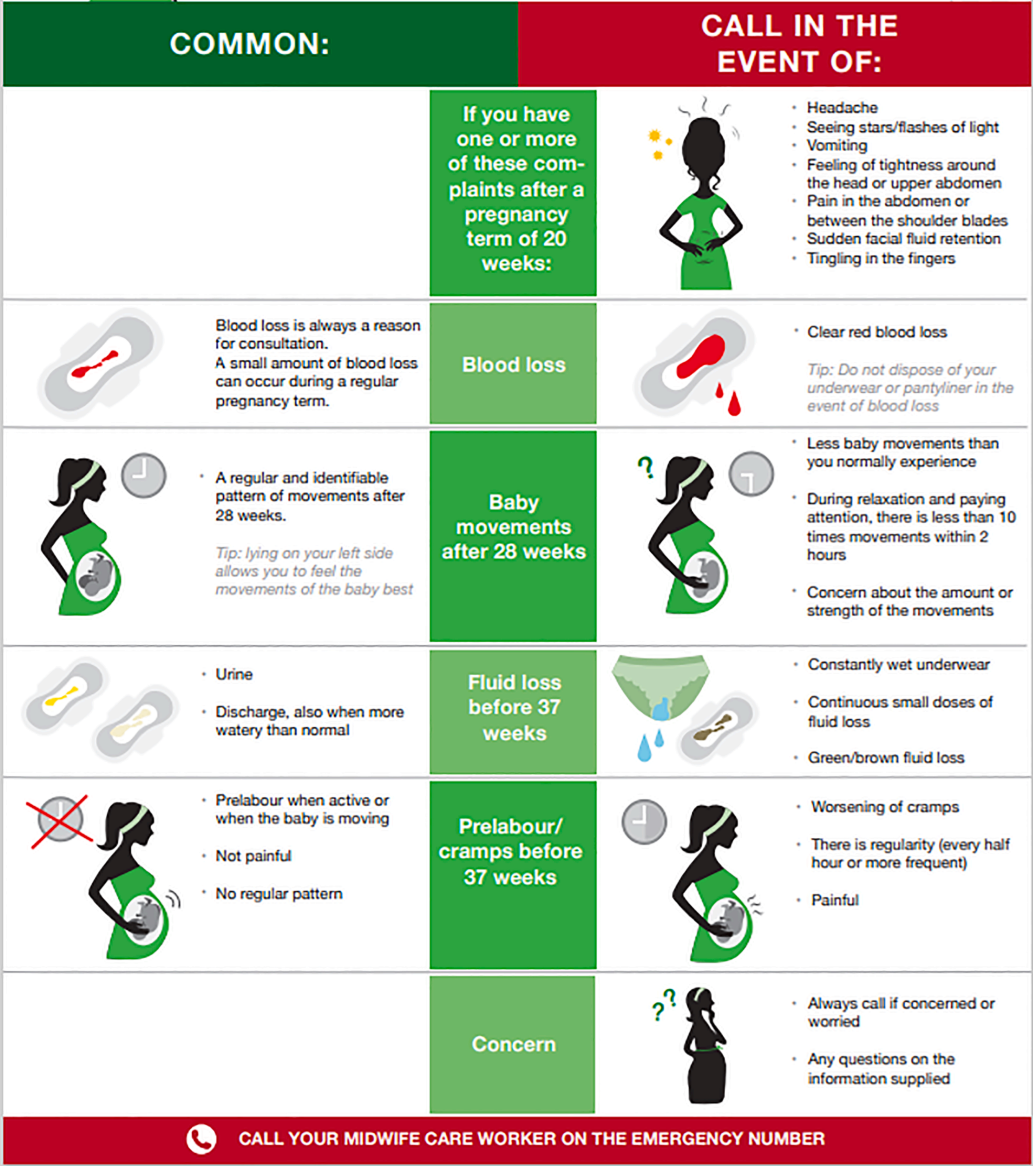


## Appendix 3 - Knowledge questionnaire

1. When you are pregnant, it is normal to experience pain during urination.

- True

- False

- I don’t know

2. A sudden increase in blood pressure can be a sign of pre-eclampsia (toxaemia of pregnancy).

- True

- False

- I don’t know

3. Children who have been breastfed after birth have a smaller chance of developing allergies than children who were not breastfed.

- True

- False

- I don’t know

4. It is safe to have sexual intercourse during pregnancy as long as there is no vaginal bleeding or fluid loss.

- True

- False

- I don’t know

5. Delivery always starts with rupture of the membranes (loss of amniotic fluid).

- True

- False

- I don’t know

6. Most of the medication you can buy without a doctor’s prescription are safe to use during pregnancy.

- True

- False

- I don’t know

7. A warm bath can alleviate the pain caused by contractions.

- True

- False

- I don’t know

8. If labour starts two weeks prior to the due date, this is called preterm labour.

- True

- False

- I don’t know

9. A baby should always be put in bed on its back, not on its side or belly.

- True

- False

- I don’t know

10. Babies of mothers who smoke during pregnancy are often smaller than babies of mothers who do not smoke during pregnancy.

- True

- False

- I don’t know

11. All pregnant women in the Netherlands pay regular visits to a gynaecologist in the hospital.

- True

- False

- I don’t know

12. If a midwife in a midwifery clinic performs my pregnancy check-ups, but I experience symptoms, I should call the hospital directly.

- True

- False

- I don’t know

13. In which of the following situations would you contact your midwife?

A. If you are worried about something

- Yes

- No

- I do not know

B. If you are experiencing vaginal bleeding

- Yes

- No

- I do not know

C. If you have a headache

- Yes

- No

- I do not know

D. If you see stars

- Yes

- No

- I do not know

E. If you have to vomit

- Yes

- No

- I do not know

F. If you experience upper abdominal pain, upper back pain or if you have the feeling of having a tight band around the upper abdomen.

- Yes

- No

- I do not know

G. If you feel the baby move less often or differently than before

- Yes

- No

- I do not know

H. If you are less than 37 weeks pregnant and you experience abdominal pain or back pain that comes and goes

- Yes

- No

- I do not know

I. If you experience loss of amniotic fluid

- Yes

- No

- I do not know

## Appendix 4: LADY-X questionnaire

**Questions about pregnancy**

1. Availability of expert healthcare professionals during pregnancy

- I felt that expert healthcare professionals were available at all times when necessary

- I felt that expert healthcare professionals were available most of the times when necessary

- I felt that expert healthcare professionals were rarely available when necessary

2. Information given by the healthcare professionals during pregnancy

- I felt very well informed by the healthcare professionals

- I felt adequately informed by the healthcare professionals

- I felt inadequately informed by the healthcare professionals

3. Taking your wishes seriously during pregnancy

- I felt that the healthcare professionals took my wishes very seriously

- I felt that the healthcare professionals took my wishes sufficiently seriously

- I felt that the healthcare professionals took my wishes insufficiently seriously

4. Emotional support by healthcare professionals during pregnancy

- I felt very well supported by the healthcare professionals

- I felt adequately supported by the healthcare professionals

- I felt inadequately supported by the healthcare professionals

5. Worries about the health of your child during pregnancy

- I was never worried about my child’s health

- I was sometimes worried about my child’s health

- I was often worried about my child’s health

Questions about childbirth

1. Presence of expert healthcare professionals during childbirth

- I felt that expert healthcare professionals were present at all times when necessary

- I felt that expert healthcare professionals were present most of the times when necessary

- I felt that expert healthcare professionals were rarely present when necessary

2. Information given by the healthcare professionals during childbirth

- I felt very well informed by the healthcare professionals

- I felt adequately informed by the healthcare professionals

- I felt inadequately informed by the healthcare professionals

3. Taking your wishes seriously during childbirth

- I felt that the healthcare professionals took my wishes very seriously

- I felt that the healthcare professionals took my wishes sufficiently seriously

- I felt that the healthcare professionals took my wishes insufficiently seriously

4. Emotional support by healthcare professionals during childbirth

- I felt very well supported by the healthcare professionals

- I felt adequately supported by the healthcare professionals

- I felt inadequately supported by the healthcare professionals

5. Feeling of security during childbirth

- I felt very safe

- I felt sufficiently safe

- I felt insufficiently safe

6. Worries about the health of your child during childbirth

- I was not worried about my child’s health

- I was somewhat worried about my child’s health

- I was very worried about my child’s health

7. Time until the first contact with your child

- Given the situation, I found that it did not take long until I had contact with my child for the first time

- Given the situation, I found that it took quite a long time until I had contact with my child for the first time

- Given the situation, I found that it took a very long time until I had contact with my child for the first time

## Footnotes

1 This manuscript focuses on pregnant asylum seekers. However, as the literature is scarce on this specific population, we sometimes refer to studies on pregnant refugees or migrants. The definitions of these populations can be found in Box 1.

## References

[bib0001] Beaton D.E., Bombardier C., Guillemin F., Ferraz M.B. (2000). Guidelines for the process of cross-cultural adaptation of self-report measures. Spine.

[bib0002] CenteringZorg. (n.d.). Project Eritrese zwangeren en moeders. Retrieved April 15, 2025, from https://centeringzorg.nl/meercentering/project-eritrese-zwangeren-2/.

[bib0003] Centraal Bureau voor de Statistiek. (2019, 8 oktober). Hoeveel asielzoekers komen naar Nederland? CBS. https://www.cbs.nl/nl-nl/dossier/dossier-asiel-migratie-en-integratie/hoeveel-asielzoekers-komen-naar-nederland.

[bib0004] Cummins A., Gibberd A., McLaughlin K., Foureur M. (2024). Midwifery continuity of care for women with perinatal mental health conditions: A cohort study from Australia. Birth.

[bib0005] Dijkstra I., Penders B., Horstman K. (2025). How health promotion prevents itself from tackling health inequalities: A critical analysis of Dutch health promotion’s paradigm through its handbooks (1995–2022). SSM – Qual. Res. Health.

[bib0006] Dokters van de Wereld, Pharos, & Nederlandse Rode Kruis. (2023, June 19). Zorgen in tijden van crisis: Gezondheidszorg in de crisisnoodopvang. https://doktersvandewereld.org/uploads/documents/Rapport-crisisnoodopvang-juni-2023_2023-06-19-145504_mlxe.pdf.

[bib0007] Dube M., Gao Y., Steel M., Bromley A., Ireland S., Kildea S. (2023). Effect of an Australian community-based caseload midwifery group practice service on maternal and neonatal outcomes for women from a refugee background. Women birth : j. Aust. Coll. Midwives.

[bib0008] von Elm E., Altman D.G., Egger M., Pocock S.J., Gøtzsche P.C., Vandenbroucke J.P., STROBE Initiative (2007). The Strengthening the Reporting of Observational Studies in Epidemiology (STROBE) statement: Guidelines for reporting observational studies. J. Clin. Epidemiol..

[bib0009] Ether S.T., Afrin S., Habib N.N., Akter F., Chowdhury A.T., Sayeed A., Raza S., Ahmed A., Saif-Ur-Rahman K.M. (2024). Managing pre and postpartum mental health issues of refugee women from fragile and conflict-affected countries: A systematic review. Public health pract. (Oxf. Engl.).

[bib0010] Feijen-de Jong E.I., Jansen D.E., Baarveld F., Boerleider A.W., Spelten E., Schellevis F., Reijneveld S.A (2015). Determinants of prenatal healthcare utilisation by low-risk women: a prospective cohort study. Women birth : j. Aust. Coll. Midwives.

[bib0011] Gärtner F.R., de Miranda E., Rijnders M.E., Freeman L.M., Middeldorp J.M., Bloemenkamp K.W., Stiggelbout A.M., van den Akker-van Marle M.E. (2015). Good reliability and validity for a new utility instrument measuring the birth experience, the Labor and Delivery Index. J. clin. epidemiol..

[bib0012] Gieles N.C., Tankink J.B., van Midde M., Düker J., van der Lans P., Wessels C.M., Bloemenkamp K.W.M., Bonsel G., van den Akker T., Goosen S., Rijken M.J., Browne J.L. (2019). Maternal and perinatal outcomes of asylum seekers and undocumented migrants in Europe: a systematic review. Eur. j. public health.

[bib0013] Glasgow R.E., Harden S.M., Gaglio B., Rabin B., Smith M.L., Porter G.C., Ory M.G., Estabrooks P.A. (2019). RE-AIM planning and evaluation framework: Adapting to new science and practice with a 20-year review. Front. Public Health.

[bib0014] Heslehurst N., Brown H., Pemu A. (2018). Perinatal health outcomes and care among asylum seekers and refugees: a systematic review of systematic reviews. BMC Med.

[bib0015] Hetherington E., Tough S., McNeil D. (2018). Vulnerable Women’s Perceptions of Individual Versus Group Prenatal Care: Results of a Cross-Sectional Survey. Matern Child Health J.

[bib0016] Hong K., Hwang H., Han H., Chae J., Choi J., Jeong Y., Lee J., Lee K.J. (2021). Perspectives on antenatal education associated with pregnancy outcomes: Systematic review and meta-analysis. Women birth : j. Aust. Coll. Midwives.

[bib0017] Inspectie Gezondheidszorg en Jeugd. (2022, August 3). Medische zorg in crisisnoodopvang asielzoekers onder enorme druk. https://www.igj.nl/actueel/nieuws/2022/08/03/medische-zorg-in-crisisnoodopvang-asielzoekers-onder-enorme-druk.

[bib0018] Jonkers M., Richters A., Zwart J., Öry F., van Roosmalen J. (2011). Severe maternal morbidity among immigrant women in the Netherlands: patients’ perspectives. Reprod. Health Matters.

[bib0019] Kasper A., Mohwinkel L.M., Nowak A.C., Kolip P. (2022). Maternal healthcare for refugee women - A qualitative review. Midwifery.

[bib0020] Koninklijke Nederlandse Organisatie van Verloskundigen. (n.d.). Kennis & Scholing. Retrieved April 15, 2025, from https://www.knov.nl/kennis-en-scholing.

[bib0021] Liu R., Chao M.T., Jostad-Laswell A., Duncan L.G. (2017). Does CenteringPregnancy Group Prenatal Care Affect the Birth Experience of Underserved Women? A Mixed Methods Analysis. J. immigr. minor. health.

[bib0022] Madeira A.D., Rangen C.M., Avery M.D. (2019). Design and Implementation of a Group Prenatal Care Model for Somali Women at a Low-Resource Health Clinic. Nurs. women's health.

[bib47] Overtoom E., Goodarzi B., Kanu S. (2026). You think, like, you’re neutral but you’re not”: a mixed- methods study of racial/ethnic bias in pain assessment, management and treatment in maternal and newborn care in the Netherlands. Int J Equity Health.

[bib0023] Owens C., Dandy J., Hancock P. (2016). Perceptions of pregnancy experiences when using a community-based antenatal service: a qualitative study of refugee and migrant women in Perth, Western Australia. Women Birth : j. Aust. Coll. Midwives.

[bib0024] van Oostrum I.E., Goosen S., Uitenbroek D.G., Koppenaal H., Stronks K. (2011). Mortality and causes of death among asylum seekers in the Netherlands, 2002-2005. J. epidemiol. community health.

[bib0025] Pereira Gray D.J., Sidaway-Lee K., White E., Thorne A., Evans P.H. (2018). Continuity of care with doctors-a matter of life and death? A systematic review of continuity of care and mortality. BMJ open.

[bib0026] Rayment-Jones H., Dalrymple K., Harris J., Harden A., Parslow E., Georgi T., Sandall J. (2021). Project20: Does continuity of care and community-based antenatal care improve maternal and neonatal birth outcomes for women with social risk factors? A prospective, observational study. PloS one.

[bib0027] Reavy K., Hobbs J., Hereford M., Crosby K. (2012). A new clinic model for refugee healthcare: Adaptation of cultural safety. Rural Remote Health.

[bib0028] Riggs E., Yelland J., Mensah F.K. (2021). Group Pregnancy Care for refugee background women: a codesigned, multimethod evaluation protocol applying a community engagement framework and an interrupted time series designBMJ. Open.

[bib0029] Rijnders M., Jans S., Aalhuizen I., Detmar S., Crone M. (2019). Women-centered care: Implementation of CenteringPregnancy® in The Netherlands. Birth (Berkeley Calif,).

[bib0030] Rotundo G. (2011). Centering pregnancy: the benefits of group prenatal care. Nurs. women's health.

[bib0031] Rowe A., Bhardwaj M., McCauley M. (2023). Maternal multimorbidity - experiences of women seeking asylum during pregnancy and after childbirth: a qualitative study. BMC Pregnancy Childbirth.

[bib0032] Sadiku F., Bucinca H., Talrich F., Molliqaj V., Selmani E., McCourt C., Rijnders M., Little G., Goodman D.C., Rising S.S., Hoxha I. (2023). Maternal satisfaction with group care: a systematic review. AJOG glob. rep..

[bib0033] Stevenson K., Edwards S., Ogunlana K., Alomari M., Agoropopoola R., Henderson W., Clemente N.S., Rayment-Jones H., McGranahan M., Castaner M.M., Luchenski S., Fellmeth G., Stevenson F., Knight M., Aldridge R. (2024). Public health, policy, and clinical interventions to improve perinatal care for migrant women and infants in high-income countries: a systematic review. EClinicalMedicine.

[bib0034] Savas Sibel T. (2024). Migrant-sensitive healthcare in Europe: advancing health equity through accessibility, acceptability, quality, and trust. Lancet Reg. Health – Eur..

[bib0035] Tankink J.B., Verschuuren A.E.H., Postma I.R., van der Lans P.J.A., de Graaf J.P., Stekelenburg J., Mesman A.W. (2021). Childbirths and the Prevalence of Potential Risk Factors for Adverse Perinatal Outcomes among Asylum Seekers in The Netherlands: A Five-Year Cross-Sectional Study. Int. j. environ. res. public health.

[bib0036] Torres M.E., Julie S., Kathryn J.L., Gwyn R.-R. (2012). Reducing maternal and child health disparities among Latino immigrants in South Carolina through a tailored, culturally appropriate and Participant-Driven initiative. Calif. J. Health Promot..

[bib0037] UN General Assembly (1951). https://www.refworld.org/docid/3be01b964.html.

[bib0038] UNHCR (2006) The Global Report 2005. UNHCR https://www.unhcr.org/publications/fundraising/4a0c04f96/global-report-2005.html.

[bib0039] United Nations. (n.d.). Definitions. Together. https://together.un.org/definitions.

[bib0040] Verschuuren A.E.H., Postma I.R., Riksen Z.M., Nott R.L., Feijen-de Jong E.I., Stekelenburg J. (2020). Pregnancy outcomes in asylum seekers in the North of the Netherlands: a retrospective documentary analysis. BMC pregnancy childbirth.

[bib0041] VluchtelingenWerk Nederland. (2023, August). Gevlucht en vergeten? Leefomstandigheden in de (crisis)noodopvang voor asielzoekers. https://www.vluchtelingenwerk.nl/sites/default/files/2023-09/Leefomstandigheden%20in%20de%20crisisnoodopvang%202023_0.pdf.

[bib0042] Wagijo M.R., Crone M.R., van Zwicht B.S., van Lith J.M.M., Schindler Rising S., Rijnders M.E.B. (2022). CenteringPregnancy in the Netherlands: Who engages, who doesn't, and why. Birth (Berkeley Calif,).

[bib0043] Wagijo M.A., Crone M., Zwicht B.B., van Lith J., Billings D.L., Rijnders M. (2023). Contributions of CenteringPregnancy to women's health behaviours, health literacy, and healthcare use in the Netherlands. Prev. med. rep..

[bib0044] Wagijo M.A., Crone M., Bruinsma-van Zwicht B., van Lith J., Billings D., Rijnders M. (2024). The effect of centeringpregnancy group antenatal care on maternal, birth, and neonatal outcomes among low-risk women in the netherlands: a stepped-wedge cluster randomized trial. J. midwifery women's health.

[bib0045] Yeshitila Y.G., Gold L., Abimanyi-Ochom J., Riggs E., Tolossa T., Le H.N.D. (2024). Effectiveness and cost-effectiveness of models of maternity care for women from migrant and refugee backgrounds in high-income countries: A systematic review. Soc. sci. med..

[bib0046] van der Zande I.S.E., van der Graaf R., Oudijk M.A., van Delden J.J.M. (2017). Vulnerability of pregnant women in clinical research. J. med. ethics.

